# Interactions between astrocytes and extracellular matrix structures contribute to neuroinflammation-associated epilepsy pathology

**DOI:** 10.3389/fmmed.2023.1198021

**Published:** 2023-06-14

**Authors:** AnnaLin M. Woo, Harald Sontheimer

**Affiliations:** ^1^ Neuroscience Graduate Program, Neuroscience Department, University of Virginia, Charlottesville, VA, United States; ^2^ Neuroscience Department, University of Virginia, Charlottesville, VA, United States

**Keywords:** astrocytes, neuroinflammation, extracellular matrix, perineuronal nets (PNNs), epilepsy, epileptogenesis, neurodegenerative disease

## Abstract

Often considered the “housekeeping” cells of the brain, astrocytes have of late been rising to the forefront of neurodegenerative disorder research. Identified as crucial components of a healthy brain, it is undeniable that when astrocytes are dysfunctional, the entire brain is thrown into disarray. We offer epilepsy as a well-studied neurological disorder in which there is clear evidence of astrocyte contribution to diseases as evidenced across several different disease models, including mouse models of hippocampal sclerosis, trauma associated epilepsy, glioma-associated epilepsy, and beta-1 integrin knockout astrogliosis. In this review we suggest that astrocyte-driven neuroinflammation, which plays a large role in the pathology of epilepsy, is at least partially modulated by interactions with perineuronal nets (PNNs), highly structured formations of the extracellular matrix (ECM). These matrix structures affect synaptic placement, but also intrinsic neuronal properties such as membrane capacitance, as well as ion buffering in their immediate milieu all of which alters neuronal excitability. We propose that the interactions between PNNs and astrocytes contribute to the disease progression of epilepsy vis a vis neuroinflammation. Further investigation and alteration of these interactions to reduce the resultant neuroinflammation may serve as a potential therapeutic target that provides an alternative to the standard anti-seizure medications from which patients are so frequently unable to benefit.

## 1 Introduction

### 1.1 Epilepsy

Affecting approximately 50 million people (World Health Organization, 2019), epilepsy is one of the most common neurological disorders in the world. Epilepsy is characterized by an individual suffering from repeated unprovoked seizures, which are a result of synchronous discharge of thousands of neurons which give rise to an abnormal EEG and are associated with a variety of behavioral abnormalities.

Temporal lobe epilepsy (TLE), which designates seizures originating in the temporal lobe, is the most common form of epilepsy observed in adults and adolescents (Blair, 2012). The majority of patients with TLE have seizures originating from internal structures of the region, which is further classified as mesial TLE (MTLE). It is well characterized by the pathological hallmark of mesial temporal lobe or hippocampal sclerosis (HS), which involves clearly demarcated regions of neuronal loss and reactivity of glial cells, or gliosis, throughout the subfields of the hippocampus and surrounding areas (Thom, 2014). Approximately one-third of patients with epilepsy are treatment-resistant, with MTLE-HS patients making up the majority of those, highlighting the need for therapeutic treatments that can specifically address the neuronal loss and gliosis that characterize HS. Although the roles of neurons and the consequences of their loss are crucial to understanding the progression of epilepsy, it has become quite evident that glial cells, particularly reactive astrocytes, can contribute to epileptogenesis; that is, the processes occurring in the brain that lead to seizures and subsequent epilepsy.

### 1.2 Astrocytes

Astrocytes have long been acknowledged as essential for normal brain function as well as being major contributors to injury and diseases. These specialized glial cells tile the entire brain and contact vasculature, synapses, and each other, forming gap junctions between the individual cells. In the healthy brain, these interactions enable astrocytes to be engaged in energy metabolism, blood-brain-barrier maintenance, glutamate clearance, and neurotransmitter uptake and homeostasis.

At the synaptic level, astrocytes effectuate not only neurotransmitter regulation but also synaptic formation, maturation, pruning, and stability (Dityatev and Schachner, 2003; Dityatev and Rusakov, 2011; Chung et al., 2015; Hösli et al., 2022). They do so by extending branching processes with small terminal extensions, often called leaflets, to contact and stabilize pre and postsynaptic partners (Khakh and Sofroniew, 2015; Torres-Ceja and Olsen, 2022), resulting in the classic “tripartite synapse.” The presence of astrocytic leaflets, which contain a variety of membrane receptors, permits astrocytes to closely monitor and respond to molecular changes in their immediate domains. These are crucial in regulating and redistributing molecules associated with neuronal firing released into the extracellular space (ECS), particularly potassium and glutamate.

Astrocytic processes are highly enriched in potassium (K^+^) channels, enabling them to clear K^+^ from the synaptic cleft and surrounding area following neuronal activity. Under normal homeostatic conditions, their inwardly rectifying potassium channels (Kir) maintain a membrane potential that hovers around the equilibrium potential for K^+^, so that upon K^+^ concentration increase in the ECS, astrocytes are able to swiftly take up excess K^+^ ions. They are then conveyed via K^+^-permeable gap junctions to neighboring astrocytes, enabling them to redistribute ions from regions of high to low K^+^ concentration (Olsen and Sontheimer, 2008; Blutstein and Haydon, 2014; Ohno, 2018). A similarly astrocyte-driven mechanism controls the extracellular concentration of the excitatory neurotransmitter glutamate, which influences neuronal excitability and can become excitotoxic if allowed to remain in the ECS. Astrocyte-specific glutamate transporters EAAT1 (GLAST) and EAAT2 (GLT-1) transport glutamate into the astrocyte along with 3Na^+^ in exchange for 1K^+^, after which the glutamate is converted to glutamine by glutamine synthetase (GS) and shuttled back to the neurons.

At each step of these synaptic processes, astrocytes inherently alter and are altered by their interactions with not only neurons, but also immune cells, signaling molecules, and even non-cellular components of the brain such as extracellular matrix. In pathological states, astrocytes can easily become reactive and transition to an inflammatory state, altering their interactions with the other brain constituents and potentially creating neuroinflammatory feedback loops.

## 2 Neuroinflammation

Neuroinflammation, which refers broadly to the innate immune response of the entire CNS, involves a non-specific immune system response to trauma, infection, disease, or other injurious challenge. This innate response of the CNS consists of a number of well-characterized responses including activation of microglia and increased production of cytokines, chemokines, antibodies, and other inflammatory molecules and mediators. Neuroinflammation can of course be beneficial by addressing and resolving the injury; alternatively, it can lead to dysfunction in the organism, dependent on what specific cytokines and chemokines are expressed and how long the tissue and cells are exposed to the signaling molecules. The main glial responders in the brain are microglia; however, astrocytes are also strongly associated with neuroinflammation and the inflammatory response, and in fact exhibit some of the swiftest inflammatory reactions following a brain injury.

### 2.1 Reactive astrocytes and astrocytic dysfunction in disease

In a neuroinflammatory situation, astrocytes can very quickly become reactive. Also referred to as “astrogliosis,” “astrocytosis,” “gliosis,” or “reactive gliosis,” these astrocytes undergo molecular, chemical, morphological, proliferative, and functional changes following an immune challenge (Sofroniew and Vinters, 2010; Vezzani et al., 2011; Zamanian et al., 2012; Robel et al., 2015; Escartin et al., 2019). These changes vary in degree of reactivity depending on the intensity or nature of the initial instigator; in fact, the heterogeneity of astrocytic responses seems to be the one agreed-upon facet of this widespread immune reaction (Escartin et al., 2019). Some reactive astrocytes are considered more beneficial or neuroprotective as they release more anti-inflammatory and health-associated signaling molecules, and others are considered more harmful or neurodegenerative as they release more pro-inflammatory, disease-associated molecules like cytokines and chemokines. This has led to a classic “good vs. bad” taxonomy of reactive astrocytes that some consider too disparate. An excellent consensus paper covers this topic (Escartin et al., 2021). For this paper, it is sufficient to express that reactive astrocytes exist along a spectrum and a single astrocyte can express both beneficial and detrimental growth factors and signaling molecules.

Keeping in mind their clearly important roles in supporting normal brain function, astrocytic dysfunction is linked to many pathologies that involve neurodegeneration including Alzheimer disease (AD) (Nwaobi et al., 2016; Pajarillo et al., 2019), Huntington’s disease (HD) (Tong et al., 2014), and amyotrophic lateral sclerosis (ALS) (Rossi et al., 2008; Ferrer, 2017; Neal and Richardson, 2018). Astrocytes and astrogliosis are also heavily implicated in epilepsy and epileptogenesis, as evidenced in the brains of human epilepsy patients (Lee et al., 1995; Crespel et al., 2002; Wetherington et al., 2008; Das et al., 2012; Devinsky et al., 2013; Eid et al., 2013; Gibbons et al., 2013; Bedner et al., 2015; Coulter and Steinhaeuser, 2015; Hayatdavoudi et al., 2022), and recapitulated in a variety of animal models.

### 2.2 Neuroinflammation in epilepsy

Neuroinflammation and its associated changes have been found in practically every neurodegenerative disorder (Escartin et al., 2019). Many studies have linked neuroinflammation with epilepsy in human patients (Ravizza et al., 2008; Aronica et al., 2012; Gibbons et al., 2013; Bedner et al., 2015; Ferrer, 2017; DeSena et al., 2018; Wenzel et al., 2019; Tan et al., 2021; Aulická et al., 2022), which has been replicated by a variety of animal epilepsy models including but not limited to: traumatic brain injury (TBI) associated epilepsy (Abdul-Muneer et al., 2016; Kim et al., 2016; Webster et al., 2017; Sharma et al., 2019; Zhou et al., 2020; Gao et al., 2022; Golub and Reddy, 2022), post-ischemic stroke epilepsy (Tröscher et al., 2021), glioma-associated epilepsy (Olsen and Sontheimer, 2008; Buckingham et al., 2011; Buckingham and Robel, 2013; MacKenzie et al., 2016; Tewari et al., 2018; Campbell et al., 2020; Komiyama, 2022), kainic acid (KA)-induced epilepsy (Canto et al., 2022; Han et al., 2019; Huang et al., 2022; Hubbard et al., 2016; McRae et al., 2010; Takahashi et al., 2010; Wolinski et al., 2022; Wu, Z et al., 2021), pilocarpine-induced epilepsy (Borges et al., 2003; Canto et al., 2022; Han et al., 2019; Kong et al., 2012; Mátyás, A et al., 2021; Ravizza et al., 2008; Schauwecker, 2012; Shapiro et al., 2008; Wyeth et al., 2012), kindling models of epilepsy (Kołosowska et al., 2016; Ueno et al., 2020), and a β1-integrin knockout astrogliosis mouse model (Robel et al., 2015). The models particularly analogous to human MTLE-HS include the pilocarpine model and the KA model, which exhibit varying degrees of HS in addition to upregulation of proteins associated with immune responses and inflammation (Canto et al., 2022).

Notably, both short-term and chronic exposure to inflammation can increase brain excitability and lead to lower seizure thresholds (Inyushin et al., 2010; Vezzani et al., 2013). In fact, application of lipopolysaccharide (LPS) to induce neuroinflammation in rat models of epilepsy has been shown to increase susceptibility to KA, pilocarpine, and pentylenetetrazol (PTZ)-induced seizures, as well as increased hippocampus neuronal degeneration (Galic et al., 2008; Huang et al., 2022).

#### 2.2.1 Specific inflammatory molecules in epilepsy

Some of the specific neuroinflammatory pathways and signals that are particularly tied to epileptic activity and epileptogenesis include cytokines such as interleukin-1β (IL-1β) (Balosso et al., 2008; Sinha et al., 2008; Maroso et al., 2010; Arisi et al., 2015; Kołosowska et al., 2016; Semple et al., 2017; Webster et al., 2017; Soltani Khaboushan et al., 2022; Zhang, 2022), the TGF-β pathway (Ivens et al., 2007; Lachos et al., 2011; Das et al., 2012; Mercado-Gómez et al., 2014; Levy et al., 2015; Kim et al., 2017), high mobility group protein B1 (HMGB1) (Maroso et al., 2010; Zurolo et al., 2012; Balosso et al., 2014; Webster et al., 2017; Zaben et al., 2021; Zhang, 2022), and tumor necrosis factor α (TNF-α) (Galic et al., 2008; Soltani Khaboushan et al., 2022), as well as chemokine C-C motif ligands 2, 3, 4, and 5 (CCL2-5) (Wu et al., 2008; Fabene et al., 2010; Kan et al., 2012; Arisi et al., 2015; Srivastava et al., 2017; Wolinski et al., 2022).

##### 2.2.1.1 IL-1β

The cytokine interleukin-1β (IL-1β) is considered to be a pro-inflammatory and has a variety of inflammation-associated downstream effectors including some of those mentioned above such as TNF-α and IL-6 (Vezzani et al., 2008). Increases or overexpression in IL-1β have been found in human patients with TLE (Zaben et al., 2021), HS and cortical dysplasia tissue (Srivastava et al., 2017), TBI associated epilepsy (Webster et al., 2017), and tumor associated epilepsy (Sun et al., 2022). This has been recapitulated in experimental epilepsy models including KA (Balosso et al., 2008; Tian et al., 2017; Wolinski et al., 2022), pilocarpine (Arisi et al., 2015), and electrical stimulation (De Simoni et al., 2000). In an epileptic setting, IL-1β is considered to be primarily secreted by activated astrocytes and microglia (Maroso et al., 2011); its receptor IL-1R1 is furthermore overexpressed in epileptic neurons and glia (Ravizza et al., 2008). Application of its endogenous antagonist IL-1Ra acts as an anticonvulsant in mice (Vezzani et al., 2000); thus, IL-1β itself may be considered a proconvulsant (Vezzani et al., 2008), although it also mediates other cell signaling pathways.

##### 2.2.1.2 TGF-β

Transforming growth factor-β (TGF-β), a family of hormonal polypeptides, is well associated with tissue homeostasis, development, and remodeling (Massagué and Chen, 2000; Stewart et al., 2018) as well as inflammation and immune modulation. The first step in a pathway with highly variable outcomes, the members of the TGF-β family function by activating Smad proteins which enter the nucleus to regulate target genes.

Activation of TGF-β signaling is associated with epilepsy (Mercado-Gómez et al., 2014; Kim et al., 2017; Webster et al., 2017), particularly when triggered by expression of extravascular albumin, i.e., in event of blood-brain barrier (BBB) leakage (Ivens et al., 2007; Webster et al., 2017). Notably, increase in albumin uptake by astrocytes has been found to correlate with downregulation of Kir4.1 channels and reduced astrocytic buffering, further contributing to epileptiform activity (Ivens et al., 2007). Other neuroinflammatory molecules associated with epilepsy such as TLR, HMGB1, and NF-κB are also affected by TGF-β signaling (Kim et al., 2017; Webster et al., 2017).

#### 2.2.2 Astrocytes and neuroinflammation in epilepsy

Although both systemic inflammation and astrogliosis are well-correlated with increased risk of or susceptibility to seizures (Wilcox et al., 2015), astrocytic roles in the overall progression of epilepsy, and whether they play a more contributory or compensatory role, are still debated. The heterogeneity of reactive astrocytes does not easily lend itself to an answer to this question; indeed, even adjacent astrocytes exposed to the same insult may exhibit differences in reactivity (Zamanian et al., 2012). Notably, though neuroinflammation can increase seizure susceptibility, seizure activity itself can upregulate the production of inflammatory markers and mediators, thus creating a vicious epileptogenic feedback loop.

We suggest that one way to investigate the correlation between neuroinflammation and epilepsy would be to investigate astrocyte interactions with a portion of the brain that has long been considered part of the support network, much like the historical role of astrocytes- the extracellular matrix.

## 3 ECM and PNNs

Rather than being a simple fluid-filled space, the gaps between adjacent cells in the brain are occupied by extracellular matrix (ECM), a loosely organized structure comprised of a variety of proteoglycans, link proteins, and hyaluronic acid. The ECM subsists in three categories: the basement membrane, which is closely associated with vasculature and blood vessels; the interstitial matrix, which is loosely structured and more associated with support and scaffolding; and perineuronal nets, which are more structured and form in only specific regions of the brain. Although all three are important, it is the perineuronal nets, hereafter referred to as “PNNs,” which will be the main focus of this review, as they are closely associated with astrocytic leaflets.

PNNs primarily form around parvalbumin-positive (PV^+^), fast-spiking GABAergic interneurons, where they surround the soma and generally extend along the axon initial segment and other neurites (Celio and Blumcke, 1994; Härtig et al., 1999; Slaker et al., 2016). Their physical appearance has historically been likened to “armor,” “lattice,” or “netting,” from which they derive their name. They are found in a number of brain regions including the cortex with high levels of density and intensity, specifically in the somatosensory cortex, visual cortex, and whisker barrel cortex in rodents, but are also present in the amygdala, hypothalamus, basal ganglia, and cerebellum (McRae et al., 2007; Bozzelli et al., 2018). Although PNNs also condense sparsely around cells in the hippocampus, they are expressed almost exclusively around excitatory neurons in the CA2 region (Carstens et al., 2016; Lensjø et al., 2017).

### 3.1 Components

**FIGURE 1:**
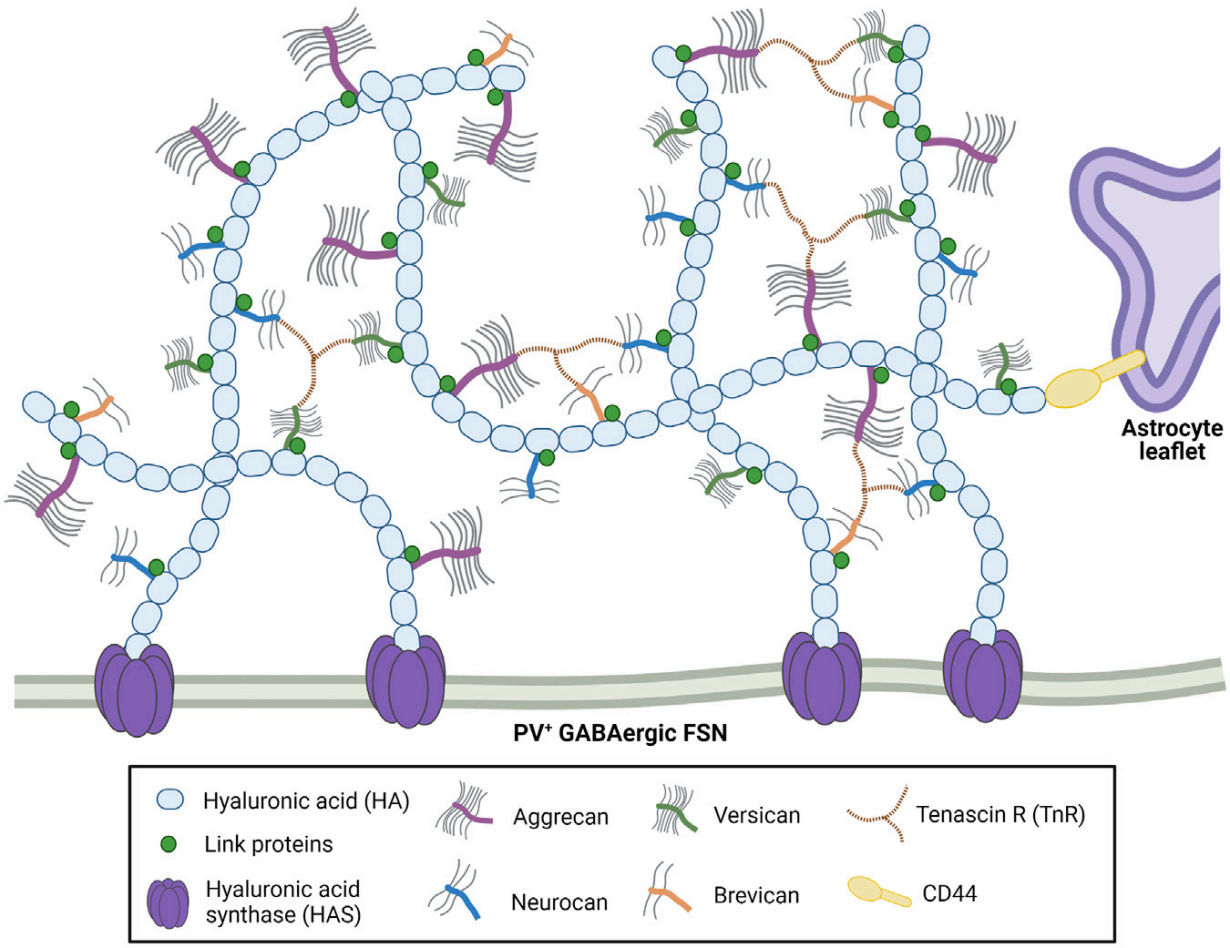
Perineuronal nets are comprised of long hyaluronic acid (HA) chains linked together with the CSPG lecticans aggrecan, versican, neurocan and brevican. Hyaluronan and proteoglycan link (Hapln) proteins and tenascin-R stabilize the CSPGs. These net-like structures are anchored by HA and hyaluronic acid synthase (HAS) on the enveloped neurons, and by HA-CD44 interactions on nearby astrocytes. Created with Biorender.com.

The molecular components of perineuronal nets are both neuronal and glial in origin (Brückner et al., 1993; Giamanco and Matthews, 2012) and include hyaluronic acid, hyaluronan and proteoglycan link (Hapln) proteins, tenascins R and C, and a variety of chondroitin sulfate proteoglycans (CSPGs), mainly of the lectican family, including aggrecan, versican, brevican, and neurocan (Figure 1).

Aggrecan is the primary lectican component of PNNs (Giamanco and Matthews, 2012; Morawski et al., 2012; Wen T. H. et al., 2018) as well as the most well-studied; it is the loss of aggrecan that is most associated with critically impaired (Kwok et al., 2010) to practically ablated (Rowlands et al., 2018) PNN structures. Aggrecan and its fellow lecticans are anchored to neuronal cell membranes by hyaluronic acid (HA), which is produced by hyaluronic acid synthase (HAS) and stabilized by Hapln proteins, predominantly Hapln1 and 4 (Kwok et al., 2010; Mohamedi et al., 2020; Jakovljević et al., 2021). HA, HAS and Hapln proteins are also critical for PNN formation (Carulli et al., 2010; Giamanco et al., 2010; Kwok et al., 2010), as is tenascin-R (TnR). TnR, which links the lecticans of the structure, is a direct component of the PNNs, whereas tenascin-C (TnC) is affiliated with the structure but does not appear to physically contribute to it (Morawski et al., 2014). Instead, it interacts with cell surface receptors like integrins and cell adhesion molecules, and indirectly modulates the other constituents of the ECM (Jakovljević et al., 2021). Aggrecan is primarily produced by neurons (Giamanco et al., 2010) and CA2 pyramidal neurons (Carstens et al., 2016), whereas most of the other CSPGs appear to be expressed by astrocytes, which express transcripts for HAPLN1, TnR, and the other three lecticans (Giamanco and Matthews, 2012). Hyaluronic acid binding protein (HABP) is associated with both neurons and glia; glial removal results in diminished but not completely depleted HABP expression *in vitro* (Giamanco and Matthews, 2012).

**FIGURE 2:**
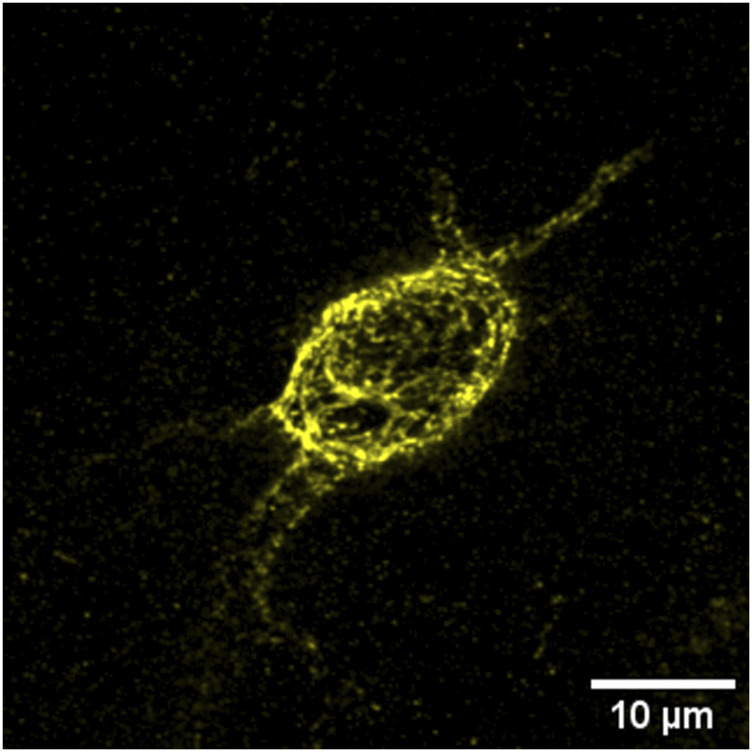
WFA^+^ PNNs (yellow) typically surround the soma and can extend along the axon initial segment and dendrites of inhibitory, parvalbumin-positive interneurons.

Visualization of these structures is most often achieved using the plant lectin marker *Wisteria floribunda agglutinin* (WFA) (Figure 2), which binds to the glycosaminoglycan (GAG) side chains of the PNNs and is considered a fairly universal marker (Giamanco et al., 2010; Slaker et al., 2016). GAGs, which adhere to the CSPG/lectican backbone of the structure, express various sulfation patterns that contribute heavily to the negative charge of PNNs as well as influencing their overall heterogeneity, dividing CSPGs into primarily two groups with either 4-sulfated or 6-sulfated GAG chains (Bonneh-Barkay, 2009; Miyata et al., 2018). These sulfation patterns- much like the nets themselves- are dynamic and have been observed to change during development, adolescence, and through adulthood (Carulli et al., 2010; Yutsudo and Kitagawa, 2015).

### 3.2 Known PNN functions

Despite being first immortalized in published form by Camillo Golgi in 1898 (Celio and Blumcke, 1994), the purposes of PNNs are still not fully elucidated. In general, the ECM is important for organization, support, and maintenance of the neural and glial cells it surrounds and encapsulates. PNNs specifically are furthermore intimately involved with the formation, stability and remodeling of synapses and synaptic signaling (Dityatev and Schachner, 2003; Frischknecht and Gundelfinger, 2012; Bozzelli et al., 2018; Lipachev et al., 2019; Reichelt et al., 2019) and thus neuronal plasticity and learning and memory (Romberg et al., 2013; Tsien, 2013; Thompson et al., 2018; Bosiacki et al., 2019; Wei et al., 2019; Chelyshev et al., 2022; Fawcett et al., 2022).

Juvenile animals still in early development display experience-dependent neuronal plasticity. This capability, observed during what is referred to as the “critical period,” is fairly depleted by the time postnatal development ends, which also coincides with the formation of PNNs (Pizzorusso et al., 2002; Hensch, 2004; Gundelfinger et al., 2010; Miyata and Kitagawa, 2015; Cornez et al., 2018). PNNs appear to stabilize or “lock” synapses into place to reduce synaptic plasticity at this point, as their physical presence restricts the placement of astrocytic leaflets and presynaptic boutons. This is particularly relevant in the sensory system, where incoming sensory information competes for cortical representation. In the visual system, for example, the closure of the ocular dominance that allocates cortical territories to each of the eyes is marked by the deposition of PNNs. Experimental degradation of the visual system PNNs using the enzymatic drug chondroitinase ABC (ChABC) reverses this process, restoring a more adolescent-like plasticity in adult animal models (Pizzorusso et al., 2002; McRae et al., 2007; Carulli et al., 2010; Hou et al., 2017). Degradation of PNNs around CA1 and CA2 hippocampal regions in mice replicated these findings, shifting the excitatory/inhibitory balance and reinstating juvenile-like plasticity (Carstens et al., 2016; Khoo et al., 2019). This characteristic stabilization of synapses further suggests a role in the formation or retention of memory (Romberg et al., 2013; Howell et al., 2015; Yang et al., 2015; Rowlands et al., 2018; Thompson et al., 2018; Wei et al., 2019) [See (Wingert and Sorg, 2021; Fawcett et al., 2022) for thorough reviews on PNNs in plasticity and memory]. Application of ChABC for treatment of glial scars, often associated with areas of neuroinflammation, was found to promote axonal regeneration and a return to plasticity in the spinal cord after injury as well (Bradbury et al., 2002; Massey et al., 2006). It is therefore proposed that PNN structures form a “repulsive barrier” that inhibits axonal and dendritic growth, not only by physically blocking leaflet and bouton formation but also via their highly negative charges as well as their interactions with growth-suppressing signaling molecules (Bonneh-Barkay, 2009; Sharma et al., 2012) [For a thorough review on glial scar formation and its immunological interactions, see (Raposo, 2014)].

Other studies have alluded to further purposes of PNNs such as helping to regulate extracellular reactive oxygen species (ROS) or protect against oxidative stress (Morawski, 2004; Beurdeley et al., 2012; Cabungcal et al., 2013) and enabling the inhibitory, GABAergic PV^+^ fast-spiking neurons (FSNs) that they envelop to fire action potentials at extremely high rates (Balmer, TS, 2016; Tewari et al., 2018). They also appear to play a role in ion buffering, as indicated by their highly anionic structures mentioned above (Brückner et al., 1993; Brückner et al., 1998; Härtig et al., 1999).

## 4 Astrocyte-ECM interactions

Astrocytes and ECM mutually interact at multiple levels in normal physiology. As discussed previously, multiple ECM components are produced by astrocytes including HAPLN1, TnR, HABP, neurocan, brevican, and versican. In the event of an injury, for example, activated astrocytes will increase secretion of CSPGs to form a glial scar around the area (Silver and Miller, 2004; Haist et al., 2012). Other ECM molecules such as TnC, laminins, and thrombospondins, while also produced by astrocytes, feature in more cell-cell signaling and cell-matrix interaction capacities instead of contributing to the physical ECM structure, and are often considered “matricellular” proteins (Eroglu, 2009; Morawski et al., 2014). Thrombospondin, for example, appears to be necessary for synaptogenesis *in vitro* and *in vivo* (Christopherson et al., 2005; Crawford et al., 2012) [See Eroglu (2009) and Jones and Bouvier (2014) for thorough reviews on astrocytically released matricellular proteins]. Astrocytes additionally are known to regulate ECM by producing molecules that degrade, remodel, and dictate the matrix structure (Kim et al., 2016), including matrix metalloproteinases and a group of metalloproteases called “a disintegrin and metalloproteinase” with and without thrombospondin motifs (ADAMs and ADAMTSs, respectively) (Ethell and Ethell, 2007; Cieplak and Strongin, 2017).

However, ECM components are also important to astrocytes, notably in development and injury response. Supplying astrocyte cultures with varying ECM proteins revealed that ECM composition determined the ability of the astrocytes to regrow following injury (Johnson et al., 2015), and alteration or removal of ECM components in development has been found to affect normal astrocytic development. Upregulation of TnC was observed post-injury (Laywell et al., 1992), as well as being associated with increased GFAP^+^ astrocytes (Karus et al., 2011). Similarly, knockout of aggrecan in chicken embryos results in altered glial precursor differentiation, favoring GFAP^+^ astrocytic cells (Domowicz et al., 2008). The glycoprotein component TnC appears to be essential for proper gliogenesis, maturation, proliferation, and differentiation (Wiese et al., 2012), with knockout resulting in changes in early astrocyte development and proliferation, and tiling *in vitro* (Ikeshima-Kataoka et al., 2007; Karus et al., 2011), and later increases in astrocytic GFAP expression *in vivo* (Karus et al., 2011).

Lastly, astrocytes also interact with the more condensed ECM structures of the brain, PNNs, on multiple levels as well. Although these have been mentioned above and will be described in later sections with greater detail, PNNs are thought to interact with astrocytes to facilitate synaptic activity, neurotransmitter uptake, and ionic buffering, all of which can be altered in inflammatory or disease states, i.e., that of the epileptic brain.

## 5 ECM and PNN alterations in epilepsy

A variety of ECM components and related molecules are altered in epilepsy. Although PNN expression varies across brain regions (Brückner et al., 1993; Yamada and Jinno, 2013), changes in the hippocampus are most often described. In human patients, studies have ranged from finding degradation of PNNs and decreases in PNN expression around PV^+^ fast-spiking neurons in chronic TLE (Perosa et al., 2002; Kim et al., 2017), to increases in diffuse ECM expression in HS(Sitaš et al., 2022) and increased expression of CSPGs and HA in MTLE hippocampus (Perosa et al., 2002). That is not to say that all individuals with epilepsy display altered brain ECM; one study even found no perceptible differences in the ECM or PNNs of adolescent or adult TLE patients (Rogers et al., 2018). However, the majority of these findings have been well replicated in the literature, primarily in rodent models of epilepsy (see Table 1).

**TABLE 1:** Alterations in ECM, PNNs, and individual PNN components in experimental epilepsy models.

Component	Changes in experimental epilepsy models	References
ECM and PNNs	↑ ECM following PTZ, KA and pilocarpine induced seizures	McRae et al. (2012), Pollock et al. (2014), Rankin-Gee et al. (2015), Yutsudo and Kitagawa (2015), Wen et al. (2018b), Han et al. (2019), Ueno et al. (2019), Ueno et al. (2020)
	↓ PNNs around PV^+^ following seizures	
CSPGs	↑ CSPG expression in epilepsy models	Naffah-Mazzacoratti et al. (1999), Yutsudo and Kitagawa (2015), Kim et al. (2017)
HA and HAS	↓ HA expression following pilocarpine seizures, TGF-β or albumin exposure	McRae et al. (2012), Arranz et al. (2014), Kim et al. (2017), Balashova et al. (2019)
	↓ expression HAS3 following pilocarpine seizures	
	HAS3 knockout mice develop SRS	
Aggrecan	↑ expression following KA seizures (transient)	McRae et al., 2012, Rankin-Gee et al. (2015), Dubey et al. (2017)
	↑ fragmentation following pilocarpine seizures	
	↓ aggrecan^+^ PNNs following pilocarpine seizures	
Neurocan and brevican	↑ brevican fragmentation following KA seizures	Yuan et al. (2002), Okamoto et al. (2003), Kim et al. (2017), Blondiaux et al. (2020)
	↓ brevican in epileptic Bassoon knockout mice	
	↑ neurocan expression following KA seizures, brain insults, TGF-β or albumin exposure	
Tenascin-C and Tenascin-R	↑ TnC following pilocarpine seizures, brain insults, TGF-β or albumin exposure	Hoffmann et al. (2009), Dityatev et al. (2010), Mercado-Gómez et al. (2014)
	TnR knockout develops kindling seizures slower than wildtype	
HAPLNs	↓ HAPLN1 expression following pilocarpine seizures, TGF-β or albumin exposure	McRae et al. (2012), Kim et al. (2017)

Abbreviations: CSPG, chondroitin sulfate proteoglycan; ECM, extracellular matrix; HA, hyaluronic acid or hyaluronan; HAS, HA synthase; Hapln, hyaluronan and proteoglycan link proteins; HS, hippocampal sclerosis; KA, kainic acid; PNN, perineuronal net; PTZ, pentylenetetrazol; PV, parvalbumin; TMEV, Theiler’s murine encephalomyelitis virus.

PNNs have generally been found to be degraded or depleted in patients and experimental models of epilepsy, whereas ECM expression overall is often similar to non-epileptic controls or may even be increased. This may be explained by several mechanisms, including but not limited to a) the fact that the most common immunohistochemical PNN marker, WFA, stains for CSPGs of the PNNs and can still mark CSPG cleavage products after degradation, or b) the possibility that astrocytes and neurons that produce ECM components ramp up production in ECM-depleting circumstances as described below.

## 6 ECM remodeling

As previously alluded to, PNNs are dynamic assemblies constantly undergoing remodeling in the healthy brain. Fluctuations in their presence, structure, density, and intensity occur during normal physiological stages from development to adulthood, mediated by the expression of remodeling enzymes that also oscillate over time and development. Recent studies also suggest seasonal behavior-based (Cornez et al., 2020; Marchand and Schwartz, 2020) and circadian or diurnal rhythm based (Pantazopoulos et al., 2020; Harkness et al., 2021) changes in PNN intensities and expression. However, loss, alteration, and malfunction of PNNs have been increasingly associated with pathological states, including trauma or injury (Lipachev et al., 2019; Mahmud et al., 2022) and a variety of psychiatric disorders (Pantazopoulos et al., 2010; De Luca and Papa, 2016; Alcaide et al., 2019; Murthy et al., 2019; Brown and Sorg, 2022). Aberrant changes in PNN expression are heavily implicated in neurodegenerative disorders as well, including AD and dementia (Baig, S et al., 2005; Crapser et al., 2020b; Logsdon et al., 2021; Yang et al., 2015), Huntington’s disease (Crapser et al., 2020a), ischemia or stroke (Härtig, 2016; Tröscher et al., 2021), and epilepsy (Kim et al., 2016; McRae et al., 2012, 2010; Rankin-Gee et al., 2015; Rogers et al., 2018; Ueno et al., 2019; Wegrzyn, D. et al., 2020).

### 6.1 MMPs and other remodeling molecules regulate ECM and inflammatory markers

#### 6.1.1 MMPs

Matrix metalloproteinases (MMPs) are a family of zinc-dependent endopeptidase enzymes expressed in and secreted by neurons, glia, and other cell types in the developing and adult nervous system (Ethell and Ethell, 2007; Reinhard et al., 2015). Once activated (by serine proteases, reactive oxygen species, nitric oxide, or other MMPs), MMPs can cleave their substrates including ECM proteins such as brevican, tenascin, aggrecan, laminin, and collagens (from which their name is derived), synaptically associated proteins such as cadherins and ephrins, growth factors and cell adhesion molecules, and cytokines such as TNF-α (Ethell and Ethell, 2007; Cieplak and Strongin, 2017). Activation of MMPs, although important in normal physiological states, is also associated with the regulation of many pathological processes, especially in the CNS wherein MMP-2, MMP-3, and MMP-9 are most abundantly found and studied. MMP-9 especially is thought to be important for brain development, critical periods, and synaptic structuring and plasticity (Reinhard et al., 2015). Notably, this MMP specifically contributes to ECM degradation following monocular deprivation (MD), leading to increased plasticity in the visual cortex which is not observed in MMP-9 knockout mice (Kelly et al., 2015; Murase et al., 2017).

Although they stimulate inflammation-associated molecules such as IL-1β and TNF-α, MMPs can also be regulated by them, including but not limited to interleukins IL-1, IL-4, and IL-6. Other enzymes, proteases and cytokines that regulate MMPs include TGF-β, TNF-α, tissue inhibitors of metalloproteinases (TIMPs), tissue plasminogen activator (tPA), and “a disintegrin and metalloproteinase” with and without thrombospondin motifs (ADAMs and ADAMTSs, respectively) (Cieplak and Strongin, 2017). TIMPs, small endogenous inhibitor proteins, can bind to and inhibit both MMPs and ADAMs/ADAMTs (Ethell and Ethell, 2007; Arpino et al., 2015). Notably, these interactions are not merely unidirectional as the ECM can also affect remodeling molecules. TGF-βs, for example- TGF-β1 in particular-are held in place in the ECM and must be released before being able to activate their signaling pathways (Hinz, 2015).

#### 6.1.2 ADAMTSs

ADAMTSs are a subgroup of cell surface metalloproteases released by neurons and glia which are associated with neurodegeneration, inflammation, adhesion to integrins, shedding of cytokines and growth factors, and degradation of ECM proteoglycans- specifically lecticans (Bonneh-Barkay, 2009; Kelwick et al., 2015; Song and Dityatev, 2018; Mohamedi et al., 2020). They themselves also regulate MMP activity, but are primarily associated with regulating ECM composition and function (Kelwick et al., 2015). ADAMTS-4 and ADAMTS-5, two of the group of ADAMTS referred to as aggrecanases or proteoglycanases, target CSPGs including aggrecan, brevican, neurocan, and versican (Nakada et al., 2005; Kelwick et al., 2015).

#### 6.1.3 tPA

tPA, another protease enzyme, activates microglia, upregulates MMP-3 and MMP-9, and promotes leakage of the BBB when activated (Shapiro, 1998; Dzwonek et al., 2004; Bonneh-Barkay, 2009; Rosenberg, 2009; Mehra et al., 2016). Increased proteolytic activity of tPA is further associated with the loss of dendritic spines in visual cortex MD; when tPA was blocked, MD associated spine loss was prevented (Mataga et al., 2004), supporting the idea that PNNs may assist in stabilizing synapses. As discussed previously, albumin leakage into the parenchyma can also trigger activation of TGF-β signaling, release of inflammatory factors such as IL-1β, and result in increased astrocytic MMP-9 levels (Ranaivo et al., 2012), which are then available to degrade ECM components and further stimulate inflammatory molecules (i.e., IL-1β). Furthermore, exposure to albumin is associated with changes in ECM components including HA, TnC, and neurocan (see Table 1).

### 6.2 Inflammatory markers associated with ECM remodeling and epilepsy

#### 6.2.1 TGF-β

Although well associated with neuroinflammation and epilepsy as covered previously, TGF-β is also known to be crucial in a number of peripheral nervous system disorders wherein tissue straining, stiffening, or scarring plays a role, including obstructive lung diseases and numerous cancers (Hinz, 2015; Chakravarthy et al., 2018; Stewart et al., 2018). Activation of TGF-β and a variety of dependent Smad proteins has been linked to ECM synthesis, remodeling, and deposition, especially in wound healing and repair (Li et al., 2003; Hinz, 2015), and increases in astrocytic TGF-β activation have been observed in PNN degradation and hyperexcitability, likely contributing to epileptogenesis (Kim et al., 2017).

#### 6.2.2 Chemokine C-C motif ligands (CCLs)

Chemokines of the CCL family, especially CCL5 and its receptor CCR5, are thought to be key in ECM regulation. A number of studies have found increased expression or upregulation of CCL5 in human epilepsy patients (Fabene et al., 2010; Srivastava et al., 2017), recapitulated in rodent models of pilocarpine (Arisi et al., 2015) and KA (Wolinski et al., 2022; Zhang et al., 2023) induced epilepsy. CCL5 has also been found to induce the expression of MMP-9 via monocytes, and is well-associated with a variety of cancers, mainly assisting in increasing MMP secretion to promote tumor invasion and dissemination (Aldinucci et al., 2020), as well as being correlated with astrocytic activation in a KA mouse model of epilepsy (Zhang et al., 2023). Experimental application of a CCL5/CCR5 antagonist was found to attenuate neuroinflammation, preventing neurodegeneration and activation of microglia (Zhang et al., 2023) and indicating the role of CCL5 in neurodegeneration in this model. Increases in another CCL, CCL2, and its corresponding receptor CCR2, have also been found in human TLE (Wu et al., 2008) as well as pilocarpine (Foresti et al., 2009; Mercado-Gómez et al., 2014; Arisi et al., 2015) and KA (Manley et al., 2007; Tian et al., 2017) induced epilepsy, where it plays a crucial role in inflammation, neuronal death, and activation of the downstream effectors STAT3 and IL-1β (Tian et al., 2017).

### 6.3 Remodeling molecules are associated with neuroinflammation and seizure activity

Though expressed and active in normal healthy physiology due to the constant turnover of ECM components, MMPs and their regulators are important mediators in CNS inflammation (Klein and Bischoff, 2011; Gaudet and Popovich, 2014) and neuroinflammatory processes (Rosenberg, 2002; Berezin et al., 2014; Reinhard et al., 2015) and have been associated with pathological disorders and diseases such as TBI(Abdul-Muneer et al., 2016; Pijet et al., 2018), stroke (Pielecka-Fortuna et al., 2015; Akol et al., 2022), glioma (Markovic et al., 2005; Varol and Sagi, 2018), and of course epilepsy (Wilczynski et al., 2008; Rankin-Gee et al., 2015; Zybura-Broda et al., 2016; Dubey et al., 2017; Pijet et al., 2018).

Systemic inflammation is well associated with increases in remodeling enzymes, especially with regards to epileptic activity. MMP-2 and MMP-9 in particular have been found to be upregulated in glia and neurons in general seizure activity, TLE, and post-status epilepticus (Dubey et al., 2017; Dzwonek et al., 2004; Kim et al., 2017; Lukasiuk et al., 2011; Pijet et al., 2018; Reinhard et al., 2015; Szklarczyk et al., 2002; Ulbrich, P. et al., 2020; Wegrzyn, D. et al., 2020; Wilczynski et al., 2008; Zybura-Broda et al., 2016). Rankin-Gee et al. (2015) find that seizure activity increases MMP proteolysis of aggrecan, which suggests a mechanism by which PNNs are degraded in epilepsy and thus contribute to the progression of the disorder. Indeed, one study found that two different strains of MMP-9 overexpressing rats displayed higher seizure susceptibility to PTZ kindling than wild type rats (Wilczynski et al., 2008). Kim et al. (2017) found upregulation of genes encoding MMP9 and 14 and ADAMTS1 in multiple brain injury and BBB leakage models as well as in resected tissue from human TLE patients (Kim et al., 2017).

In glioma-associated epilepsy, for example, epileptic activity in peritumoral areas may be attributed to MMP-driven PNN degradation. Glioma cells and tumor-associated macrophages (TAMs) release MMPs, but the host’s inflammatory cells can also release MMPs in response to the tumor cells. Glioma has also been found to overexpress ADAMTS-5, which as mentioned previously targets CSPGs, specifically cleaving brevican (Nakada et al., 2005). Furthermore, the ECM itself is thought to actively promote cancer growth by altering collagen degradation and re-deposition via remodeling enzymes so as to clear space and allow progression and growth of the tumor (Shapiro, 1998; Markovic et al., 2005; Varol and Sagi, 2018). Peritumoral areas immediately surrounding resected low-grade epilepsy-associated tumors exhibit not only increased inflammatory markers, but also an increased ripple rate, possibly implicating an MMP-driven discrepancy in excitatory-inhibitory balance (Sun et al., 2022).

Treatment of cultured rat astrocytes and microglia with inflammatory mediators such as IL-1β, TNF-α, and LPS also stimulates the production of MMP-2 and MMP-9 (Gottschall and Deb, 1996; Shapiro, 1998; Dzwonek et al., 2004), and accelerates the epileptogenesis process and/or increases seizure susceptibility in rat models of kindling induced seizures (Wilczynski et al., 2008; Kołosowska et al., 2016). In concurrence, application of MMP inhibitors or knockout of MMP-9 seemingly protected mice and rats against KA-induced and kindling-induced seizures (Wilczynski et al., 2008; Pollock et al., 2014) as well as TBI-induced spontaneous seizures (Pijet et al., 2018). Notably, a critical amount of MMPs seems to be required for optimal function-inhibiting MMP-2 and MMP-9 can suppress plasticity in the visual cortex, but briefly inhibiting the same MMPs post-stroke can rescue plasticity (Akol et al., 2022), indicating that intervention timing and intensity are crucial.

### 6.4 ECM cleavage products are associated with neuroinflammation

Degraded segments of PNNs and ECM are known to act as alarmins or damage-associated molecular patterns (DAMPs) and thus amplify CNS inflammation (Gaudet and Popovich, 2014; Jang et al., 2020). Buildup of fragmented HA in particular, specifically the low molecular weight (LMW) HA (10–500 kDa) generated due to ECM damage, is known to serve as an injury and inflammatory signal, binding to CD-44 and TLR4 to induce pathways such as NFκB signaling and increasing IL-1β and TNF-α *in vitro* (Noble, 2002; Wang et al., 2006). Hyaluronidase treatment of cultured rat astrocytes induced more stellate-like, branching morphology, indicating cleavage of HA may be associated with astrocytic form and/or function (Konopka et al., 2016).

CSPGs and tenascins are released from activated astrocytes following CNS injury, with TnC specifically increasing after exposure to IL-1β, TNF-α and INF-γ (Laywell et al., 1992; Jang et al., 2020). TnC serves as an activator of TLR4, which is well associated with increased pro-inflammatory cytokines and neuroinflammation (Midwood et al., 2009; Eidson et al., 2017). Studies have also found increased CSPG expression in the pathological hallmarks of neurodegenerative diseases with chronic inflammation components such as AD plaques and tangles and MS lesions (Jang et al., 2020).

This is not to say that all ECM components and cleavage products are pro-inflammatory; in fact, the GAG sidechains of CSPGs may have different effects due to increased or decreased affinity for specific chemokines depending on their sulfation pattern [see (Monneau et al., 2016) for a thorough review on chemokine-GAG interactions]. 6-sulfated CSPGs, for example, appear to help suppress microglial activation and production of IL-6 and TNF-α (Tan and Tabata, 2014; Jang et al., 2020).

## 7 Inflammatory astrocyte-ECM interactions contribute to epileptogenesis and epilepsy

Experimental degradation of PNNs or removal of its components can lead to increased propensity to epileptic activity, but may also in and of itself cause spontaneous seizure activity (Arranz et al., 2014; Tewari et al., 2018; Balashova et al., 2019; Wegrzyn. et al., 2020). Seizure activity, however, appears to cause degradation of ECM and PNNs (McRae et al., 2012; Pollock et al., 2014; Dubey et al., 2017; Ueno et al., 2019). Thus, one incidence of epileptogenic activity or PNN alteration could easily begin a feedback loop of increased degradation accompanied by increased seizure activity.

We suggest that some of these correlations are due to neuroinflammatory pathways triggered by changes in how PNNs and astrocytes are interacting, specifically at the levels of a) synapses, b) ionic buffering, and c) other biophysical properties such as cell membrane capacitance.

## 8 Synapses

Astrocytes and PNNs interact at the synapse in ways that may lead to neuroinflammation, thus feeding into the potential for increased susceptibility to or increased severity of epilepsy.

### 8.1 Reactive astrocytes affect ECM components

Many of the molecules released by reactive astrocytes can indirectly or directly lead to ECM-altering outcomes, including changes in the expression of HA, CSPGs, and tenascin proteins (Wiese et al., 2012; Bosiacki et al., 2019). Activated astrocytes are known to migrate to injury sites in the CNS and release inflammatory factors such as CCL2 and 3 (Fabene et al., 2010), as well as increasing secretion of CSPGs including neurocan, versican, and brevican, likely via TGF-β and subsequent signaling (Schiller et al., 2004). Genes associated with ECM and integrin signaling are also significantly upregulated in rat models of kainic acid and pilocarpine epilepsy after SE (Han et al., 2019) as well as being associated with genes upregulated in astrogliosis (Zamanian et al., 2012). PTZ-induced seizures were found to trigger astrogliosis in the targeted hippocampus and many cortical areas, as well as overall increases in the amount of extracellular matrix (Ueno et al., 2020) [For a thorough review of glial-ECM remodeling, see (Kim et al., 2016)].

### 8.2 Changes in CSPGs and tenascins alter synapses

Removal of multiple lecticans *in vivo* and *in vitro* is associated with not only abnormal PNN morphology, but also altered synaptic function, including reduced inhibitory synapses and increased excitatory presynaptic markers (Geissler et al., 2013) (Mueller-Buehl et al., 2022). Aggrecan knockout cells show a complete lack of PNNs *in vitro* (Kwok et al., 2010), but removal of other CSPGs does not appear to lead to such drastic changes. Brevican, for example, appears to be required for modulating synapses and excitatory contacts of inhibitory interneurons; lack of brevican at PV^+^ interneurons led to altered pruning of excitatory synapses and thus alterations in spike properties and miniature EPSCs (Favuzzi et al., 2017). Although a genetically deleted brevican mouse model did not show changes in the structure of the PNN itself, multiple studies reported seeing significant alterations of synaptic plasticity and transmission (Frischknecht and Gundelfinger, 2012; Blosa et al., 2016). A knockout mouse model of neurocan also correlated with notable decreases in brevican mRNA levels and visibly altered brevican ECM structures (Sonntag et al., 2018; Schmidt et al., 2020), though neurocan itself did not appear to be altered. Astrocytically released ECM molecules such as TnR and laminins interact directly with voltage-gated Ca^2^ channels, AMPARs, and GABARs, and as such, influence synaptic organization and function (Dityatev and Schachner, 2006); thus, upon astrogliosis, upregulation or alteration of ECM components and signaling molecules can easily follow.

### 8.3 Changes in CSPGs also alter neural networks

Replicating CSPG degradation along with removal of HA *in vitro* was found to increase synaptogenesis and decrease glutamate sensitivity (Pyka, 2011), both of which could readily lead to increases in excitability. Increases in excitability are not the only method by which the excitation/inhibition balance can be altered; in fact, a study in which PNNs were experimentally degraded using ChABC showed reduced excitability of PV^+^ neurons and inhibitory synaptic transmission in the visual cortex (Liu et al., 2019). Similarly, a recent study (Dzyubenko, 2021) that also experimentally degraded PNNs saw a decrease in the density of inhibitory synapses to both excitatory and inhibitory neurons, along with an increase in the strength of inhibitory synapses. However, the action potential threshold for excitatory neurons also decreased, and as such, the strengthened inhibitory neuron outputs were insufficient to balance the excitatory activity, leading to overall network changes (Dzyubenko, 2021).

### 8.4 Neuroinflammation-associated molecules regulate synapses

#### 8.4.1 IL-33

The cytokine interleukin-33 (IL-33) is well established as a mediator of ECM remodeling, provided by both neurons and astrocytes. Release of IL-33 from hippocampal neurons in an experience-dependent matter has been found to activate microglia engulfment and remodeling of ECM, thus resulting in synaptic plasticity (Yasuoka et al., 2011; Vainchtein et al., 2018; Nguyen et al., 2020). Furthermore, astrocytic IL-33 mRNA and protein results in proliferation of microglia and increased proinflammatory cytokines like IL-1β and TNF-α (Yasuoka et al., 2011).

Suppression of hippocampal neuronal activity increases astrocytic release of IL-33 and has been found to promote increased excitatory synaptogenesis (Hudson et al., 2008; Wang et al., 2020). Notably, astrocytic IL-33 expression increases upon exposure to pathogen-associated molecular patterns (PAMPs) (Hudson et al., 2008), meaning that immune activation may be associated with increased excitation. If the increased neuronal activity is then suppressed, release of IL-33 may also increase, leading to an inflammatory-synaptogenesis feedback loop which would affect not only individual neuronal activity but again, overall network changes and thus the potential for epileptic activity.

#### 8.4.2 HA-CD44 interactions

CD44 is a widely expressed transmembrane protein that serves as a receptor for HA and has been associated with cell adhesion, inflammation, and production of cytokines (Levesque and Haynes, 1997; Puré and Cuff, 2001). When expressed in myeloid cells, CD44 has been implicated in increased production of MMP-9, TNF-α, and IL-1β *in vitro* via TLR2 activation (Ivanova, 2022). However, other studies suggest an anti-inflammatory role of the CD44 receptor (Neal, 2018).

Expression of HA within the synaptic cleft decreases towards the end of postnatal development but increases around the synaptic cleft as the critical period is ending, when the formation of PNNs is being finalized (Wilson and Litwa, 2021; Allnoch et al., 2022). This increase in expression is likely due to the role of HA in anchoring the PNN structure to astrocytic leaflets at the synapse via its binding interactions with CD44 (Dzwonek et al., 2004; Carulli et al., 2006; Kwok et al., 2010; Miyata and Kitagawa, 2017; Wen et al., 2018b), indicating its importance in the stability of said synapse. Indeed, overexpression of HAS2 seems to inhibit the occurrence of spontaneous activity through synaptic HA synthesis (Wilson and Litwa, 2021), although the mechanism of how the HA is altering this is unclear. One potential process may involve overproduced HA anchoring PNN components to leaflets in an overly abundant manner, going so far as to interfere with normal synaptic function.

Conversely, a mouse knockout of HAS2 is associated with not only decreased HA levels in the cortex, but also an increase in epileptic seizures (Arranz et al., 2014; Perkins et al., 2017), implicating the loss of stable PNNs as epileptogenic. CD44 also seems to play a role, as HA-CD44 interactions can influence morphological changes in astrocytes via Rac1 signaling, providing evidence that ECM-driven alterations circle back to alter astrocytes (Konopka et al., 2016). Knockdown of CD44 in hippocampal neurons is associated with altered spine morphology and decreased functional synapses, as well as significantly decreased spontaneous excitatory activity (Roszkowska et al., 2016), again likely enhancing the instability of synapses and the lack of normal functional synapses via loss of the PNNs as a stabilizing component.

Notably AMPARs, which mediate excitatory currents, are restricted and stabilized by the presence of PNNs (Frischknecht et al., 2009). Alteration and/or destabilization of PNNs could thereby increase the mobility and exchange of AMPARs, thus altering activity at the excitatory synapses, not to mention plasticity and overall network excitability. Wilson and Litwa (Wilson and Litwa, 2021) further note that overexpression of CD44 decreases excitatory synapse formation, which aligns well with the proposed role of PNNs in anchoring AMPARs. Interestingly, blocking AMPARs after PTZ-induced seizures ameliorated seizure activity, but also greatly increased the overall levels of aggrecan, TnR, and neurocan in the brain (Chen, 2016).

### 8.5 Neuroinflammation-induced PNN changes affect astrocytes

TnC, which interacts with other cell surface receptors and helps to regulate cell growth, adhesion, and migration, is upregulated early in inflammation, either by the pro-inflammatory IL-1 pathway or possibly by IL-4, IL-13, or TGF-β, which are considered anti-inflammatory (Chiquet-Ehrismann and Chiquet, 2003). It is expressed at high concentrations in disorders characterized by chronic inflammation, and its production further induces inflammatory responses, as seen in astrocytes *in vitro* (Wiese et al., 2012) and a mouse model of AD, for example (Xie et al., 2013). TnC further appears to regulate astrocytic maturation during embryonic development in cortical cells and in the spinal cord (Karus et al., 2011; Wiese et al., 2012), implicating this ECM molecule in astrocyte development and providing a potential feedback loop effect of altered PNNs affecting astrocytes. HA also interacts with astrocytes at two specific molecular weights (low HA, 10–500 kDa and high HA, >500 kDa), both of which appear to modulate astrocytic responses to TLR agonists and upregulate IL-10 expression via TLR pathways (Chistyakov et al., 2019).

Taken together, it is clear that the presence of PNNs is well-associated with astrocytes at the synapse, and that neuroinflammation can not only alter these interactions but can be upregulated as a result of these interactions as well.

## 9 Ion buffering

Astrocytes’ maintenance of potassium (K^+^) and glutamate concentrations in the healthy brain is well established, as is their dysfunction in times of immune challenge, injury, and disease. As excessive extracellular K^+^ is associated with increased excitation and/or hyperexcitability, K^+^ spatial buffering in the healthy brain serves as a regulatory and protective necessity. Glutamate regulation by astrocytes is crucial as well, as excessive extracellular glutamate can also lead to excitotoxicity and hyperexcitability.

These regulatory processes are not just left to the astrocytes, however: PNNs also have a role in ionic buffering. One school of thought is that the strong negative charge of the structures, endowed by the negatively charged sulfated GAG side chains, enables PNNs to quickly bind up extracellular K^+^ to clear the ECS, thus preventing a buildup of excess positive charges and associated hyperexcitability, which allows the local neurons to continue firing (Brückner et al., 1993; Härtig et al., 1999).

Another is that the negative charge of the PNNs has less to do with quickly clearing the cations, but more about capturing and holding them as a type of “reservoir” to keep them readily available for altering the local ionic gradients (Morawski et al., 2015). In fact, one study proposes that the anionic charges of the ECM surrounding hippocampal neurons actually change the Cl^-^ gradient across the membrane, thereby making GABA receptors excitable (Glykys et al., 2014). It must be further noted that the acknowledged role of PNNs in maintaining extracellular space also affects diffusion of molecules within the ECS, with degradation or reduction of PNN structure contributing to increased diffusion capabilities (Syková, 2004; Morawski et al., 2015). Cations released in the somatosensory cortex and auditory cortex, which have the most dense expression of PNNs, display a more restrained pattern of diffusion than other regions, and degradation of ECM restores a more regular isotropic diffusion of the released charges (Morawski et al., 2015). The GAGs present in the PNNs and on cell surfaces additionally interact with chemokines upon inflammatory stimuli, creating the concentration gradient necessary for chemokine-induced leukocyte recruitment and migration (Crijns et al., 2020); this too can alter diffusion in the ECS.

Lastly, the concrete physical presence of the PNNs- which influences astrocytic leaflet placement, as covered in the synapse segment previously-can certainly influence the presence of leaflets and thus their ability to take up excess extracellular ions at the synapse.

As mentioned above, dysfunctional regulation of K^+^ and glutamate in reactive astrocytes is already well associated with epilepsy and hyperexcitability [see (Robel and Sontheimer, 2016) for review]. However, we propose that one of the driving forces behind epileptic activity is neuroinflammation due to astrocyte-PNN interactions that change how ions are regulated at the synapse and in the ECS.

### 9.1 Potassium and glutamate regulation is altered in epilepsy

#### 9.1.1 Potassium

Dysfunctional transportation or uptake of K^+^ is an established finding in reactive and tumor-associated astrocytes (Campbell et al., 2020), TBI-associated epilepsy (Coulter and Steinhaeuser, 2015), and MTLE-HS (Thom, 2014; Coulter and Steinhaeuser, 2015), and has been associated with hyperexcitability and epileptic activity. Reduced K^+^ buffering has been found to facilitate EPSPs (David et al., 2009; Kinboshi, M et al., 2020), which affects not only individual neuronal firing but can thus alter network excitation. This alteration is likely due to decreased Kir currents, which has been observed in many epilepsy models (Inyushin et al., 2010; Das et al., 2012; Robel et al., 2015; Nwaobi et al., 2016; Kinboshi et al., 2020; Akyuz et al., 2022). Rodent models in which Kir4.1 is specifically knocked out exhibit astrocytic membrane depolarization and subsequently dysregulated K^+^ and glutamate homeostasis, contributing to increased seizure susceptibility or activity (Djukic et al., 2007; Olsen and Sontheimer, 2008; Inyushin et al., 2010) and further solidifying Kir4.1 as an essential potassium channel in glial and neuronal homeostasis. A recent study found that KA-treated rats had an increased susceptibility to seizures via TNFα-mediated necroptosis altering BBB integrity, as well as increased levels of K^+^ and glutamate in the extracellular space (Huang et al., 2022), which could indicate dysregulation of glutamate and potassium.

Additionally, although astrocytes can take up albumin that enters the parenchyma due to damaged or otherwise altered BBB, that uptake is associated with transcriptional downregulation of Kir4.1 and Kir2.3 channels. This Kir downregulation can result in impaired gap junction coupling [associated with local inflammation (Karpuk et al., 2011)], altered potassium buffering, and hyperexcitability (David et al., 2009; Aronica et al., 2012; Coulter and Steinhaeuser, 2015), all of which are established as correlative in epileptogenesis. Notably, mRNA and protein expression of Kir4.1 appear to be at least partially regulated by cytokine activity in both human epileptic patients as well as in rodent models (Zurolo et al., 2012; Huang et al., 2022), implicating inflammation (and specifically the cytokine IL-1β) as a contributing pathway.

#### 9.1.2 Glutamate

In addition to impaired Kir channels, reactive astrocytes in the MTLE brain express altered expression of glutamine synthetase (GS), leading to impaired uptake, metabolism of, and release of glutamate (Eid et al., 2013).

Dysregulation of glutamate is associated with the breakdown of normal astrocyte function (Takahashi et al., 2010; Khakh and Sofroniew, 2015), and excessive glutamate in the ECS is a well-known feature of and contributor to epilepsy in both human patients and animal models (Buckingham et al., 2011; Eid et al., 2013; Coulter and Steinhaeuser, 2015; Robel et al., 2015; Robert et al., 2015) as well as being associated with neuroinflammation, specifically inflammatory cytokines such as IL-1β and TNF-α, which have been found to attenuate astrocytic glutamate uptake (Ye and Sontheimer, 1996) in turn.

Potassium and glutamate dysregulation are not ubiquitous in neuroinflammatory states or even in seizure disorders, however. For example, potassium currents were found to be unimpaired in a KA-induced TLE rat model which also exhibited increased gap junction coupling, and surprisingly, a more efficient transport cascade of glutamate (Takahashi et al., 2010). The authors further noted that the KA-treated rats that developed epilepsy displayed swifter synaptic glutamate clearance but no changes in GLT-1 or GLAST receptors [Notably, dysregulation associated with both upregulation and downregulation of EAAT1 and EAAT2 has been found in epilepsy (Coulter and Steinhaeuser, 2015; Hubbard et al., 2016)]. A potential mechanism could involve the degradation of ECM allowing for increased ionic diffusion as seen in the study of ECS diffusion previously mentioned (Syková, 2004). Lastly, Chaunsali et al. (2021) propose that the negative charge of PNNs plays a neuroprotective role by repelling negatively charged extracellular glutamate, thus opening neurons to glutamatergic excitotoxicity and possibly cell death when PNNs are degraded.

As PNNs are being altered, either within normal or inflammatory bounds, placement of astrocytes and thus the expression of EAAT receptors and Kir channels at the synapse are also changed. If these ECM structures are deficient or completely absent from the synapses and cannot regulate K^+^ and glutamate as per their standard role, the potential excitotoxicity of excess ionic concentrations in the ECS could easily shift the balance of not only individual neuronal excitation but overall circuit and brain excitation as well.

## 10 Membrane capacitance and intrinsic neuronal properties

Considering that PNNs preferentially surround PV^+^, GABAergic inhibitory interneurons, it is reasonable to hypothesize that altering the surrounding PNN affects the functionality or inherent capabilities and characteristics of these fast-spiking cells (Brückner et al., 1993; Härtig et al., 1999). In exploring the biophysical properties of PV^+^ FSNs, we and others were surprised to find that enzymatic removal or glioma-associated loss of PNNs altered the characteristics of the enveloped cells, resulting in decreased excitation as well as a decrease in cell membrane capacitance (Balmer, 2016; Tewari et al., 2018). This has resulted in the hypothesis that PNNs act as an insulator, reducing the specific membrane capacitance of the cell in a myelin-like manner to enable extremely high firing rates. A more recent study found that degradation of PNNs disrupts not only the PV^+^ cells themselves but also their role of stabilizing local circuits and network activity in the medial entorhinal cortex (Christensen et al., 2021). Thus, PNNs appear to be required for consistent, fast firing of inhibitory neurons, and their degradation (i.e., by astrocytically-released remodeling agents, or by changes in the primarily astrocytically-secreted ECM components such as brevican, neurocan, versican, HAPLNs, and TnR) can lead to decreased inhibition, resulting in asynchronized local network activity and overall circuit hyperexcitability.

Following these surprising findings, further exploration of the broader applicability of PNNs’ effects on firing properties and capacitance is required. For one, individual PNN constituents may have biophysical property-altering effects. An early mouse model of brevican depletion, for example, showed minimal abnormalities in PNNs but significant deficiencies in hippocampal LTP (Brakebusch et al., 2002), which may be attributed to brevican’s influence on AMPAR and K^+^ channel localization (Favuzzi et al., 2017). Altering brevican in a PV^+^ cell results in altered electrophysiological patterns, wherein its presence correlates with higher numbers of excitatory synaptic inputs, and deletion increases the intrinsic excitability of the cell by lowering the action potential threshold and decreasing latency to firing (Favuzzi et al., 2017). Interestingly, the authors additionally observed that resected human tissue from TLE patients also expressed decreased brevican levels in the cortex, further implicating loss of brevican in altered excitability in TLE.

Degradation of PNNs can alter biophysical cell properties in more than one way, however. Fragments of CSPGs, as discussed previously, can trigger immune responses, and one study found that free CS proteins can also trigger cell depolarization in rat hippocampal neurons *in vitro* via AMPA and kainate receptors (Maroto et al., 2013). The authors posit that injury or pathology-induced MMP degradation of CSPGs releases free CSs to effectuate Ca^2+^ signal via AMPARs in order to facilitate cell migratory or axonal regrowth.

PNN-enveloped PV^+^ interneurons thus appear to rely on their PNN coatings to maintain normal physiological functions including cell membrane capacitance and firing rate, not to mention ion channel and receptor localization. As such, we suggest that abnormal PNN-astrocyte interactions brought about by neuroinflammation can alter these properties, and may further induce neuroinflammatory reactions themselves, thus feeding back into an epileptogenic brain environment.

## 11 Discussion

**FIGURE 3:**
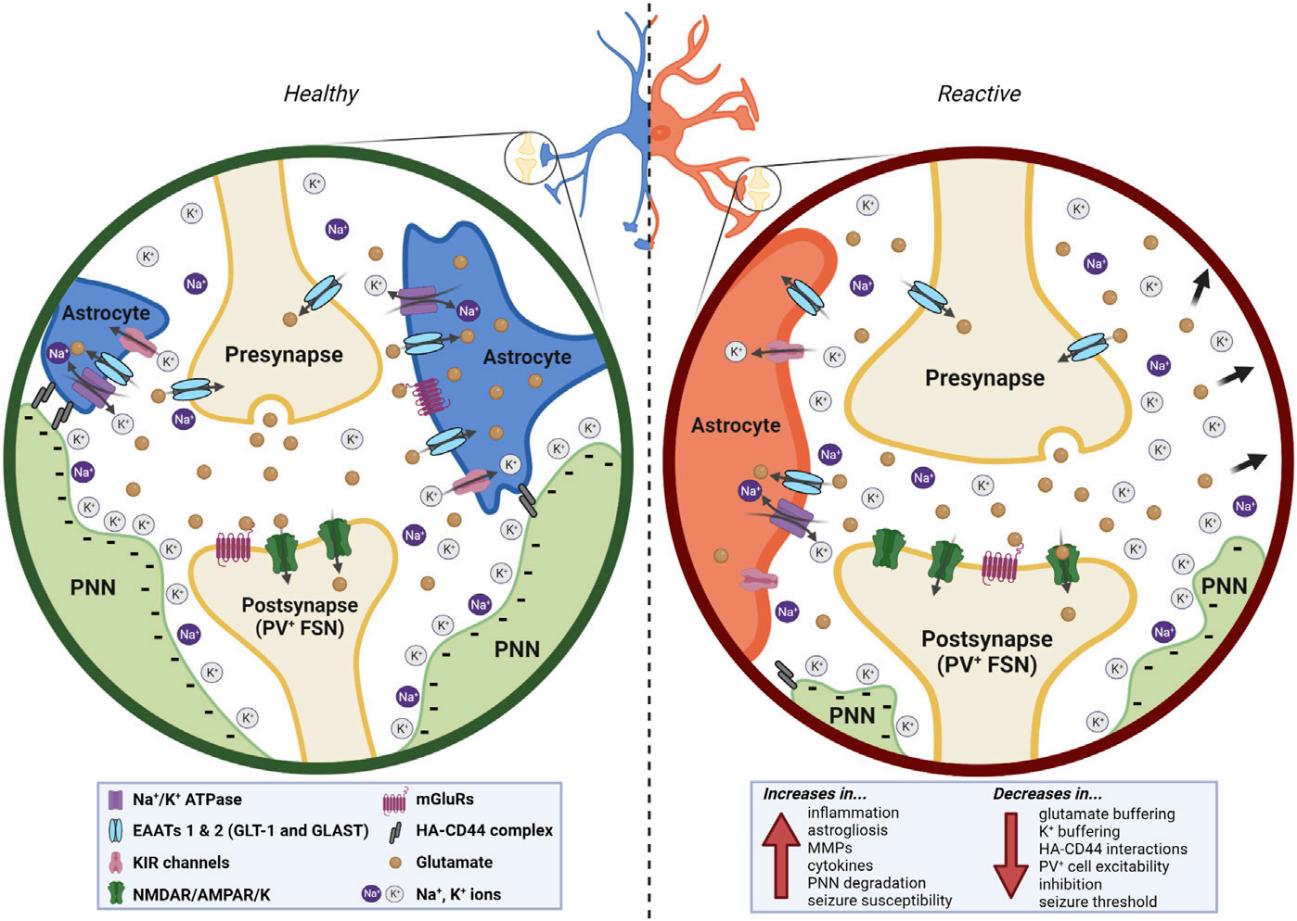
In the healthy brain (left), astrocytes and ECM interact at multiple levels. The presence of the PNN around the PV^+^ FSN postsynaptic bouton of the synapse helps to stabilize the astrocytic leaflets and serves as a highly anionic structure that redistributes and buffers cations released in and around the synaptic cleft. Glutamate, K^+^, and Na^+^ are sufficiently redistributed and taken up. The PNN additionally functions as insulation to the FSN it surrounds. In a neuroinflammatory state (right), astrocytes and PNNs interact abnormally due to the degradation of PNNs and the reactivity of the astrocytes, resulting in a variety of both upregulated and downregulated effects (bottom right) that stem from and contribute to the neuroinflammatory state. Created with Biorender.com.

Astrocytes and PNNs interact to induce and increase neuroinflammation, leading to a susceptibility to or increase in seizures and epilepsy. We have summarized three ways in which they interact, suggesting that altered synaptic placement, ionic buffering, and biophysical cellular properties such as capacitance can influence and be influenced by neuroinflammation, and thus contribute to epileptogenesis (Figure 3). This is not a completely new hypothesis; in fact, it has been proposed that even just the composition of the ECM determines astrocyte responses to mechanical and inflammatory stimuli (Johnson et al., 2015). This is not to say that neuroinflammation in and of itself is necessary and sufficient to cause epileptic activity, but we suggest it serves as a key contributor to the process. We further propose that dysfunctional interactions between PNNs and astrocytes can serve as a feedback loop, inducing and/or enhancing neuroinflammation-thus potentially acting as both a cause and consequence of epilepsy.

### 11.1 Controversies and fundamental concepts, issues, and problems

At this point, there appears general agreement that PNNs are crucial for synaptic function and retention of memory, and that removal of or otherwise diminished PNNs can be associated with a variety of brain disorders. Whether PNNs are altered in neurodegenerative disorders specifically has historically been controversial. A recent study (Crapser et al., 2020b) has provided strong evidence for microglial engulfment of PNNs in AD, further showing that induction of an inflammatory state using LPS injections induced similar PNN-degrading phenotypes in wild type mice. There have also been a number of studies looking at alterations of PNNs in psychiatric disorders in particular [see (Carceller et al., 2022) for a thorough review].

Due to the inflammation aspect of our hypothesis, although it was not discussed, other immune cells such as microglia certainly have their own interactions with astrocytes and PNNs. As mentioned above, Crapser et al. (2020b) found that activated microglia are heavily implicated in PNN degradation, whether directly or indirectly, and another 2020 study (Nguyen et al., 2020) found that cytokine IL-33 released by hippocampal neurons induces microglial ECM remodeling. Astrocytically-released IL-33 has also been found to drive synaptic engulfment by microglia (Vainchtein et al., 2018) and microglial activation has been linked to epilepsy in general (Shapiro et al., 2008; Hiragi et al., 2018) [see (Andoh and Koyama, 2021) for review of microglia and plasticity]. Another paper looking specifically at depletion of microglia in Huntington’s disease found that knockout of microglia resulted in decreased PNN degradation, with denser PNN expression in all brain regions as well as reduced astrogliosis (Crapser et al., 2020a).

The time course of all this PNN remodeling may still be up for debate as well; one study suggests PNN modification occurs during each sleep cycle, varying with circadian rhythms (Pantazopoulos et al., 2020). However, such a quick turnaround of PNN degradation and production would likely have larger implications in multiple disease states as well as in healthy brains, where again, PNNs appear to play critical roles in plasticity and stability and are required for the normal function of the enveloped PV^+^ FSNs. A more recent study observed that although PNN expression did not change diurnally, it does increase in the absence of microglia, which display changes in ramification during the circadian cycle in mice (Barahona et al., 2022). If PNN integrity does in fact alter every 24-h cycle, the question of how normal brain function is maintained-especially with regards to inhibitory neuronal activity from FSNs-comes to the forefront.

Lastly, although touched upon earlier, the physiology of reactive astrocytes and the classification of such has continued to be controversial. Formerly considered in more binary terms such as “reactive” versus “nonreactive,” or “neuroprotective” vs. “neurotoxic,” astrocytes are now more likely to be classified holistically and along a continuum, categorized by their morphological, functional, and molecular changes, as well as taking into consideration their immunoreactivity markers and the brain regions they are expressed in, amongst others factors (Escartin et al., 2021).

### 11.2 Current research gaps and potential developments in the field

To fully explore this hypothesis, designing experiments to artificially alter the proposed PNN-astrocyte interactions is the crucial next step. Although there are enzymes that can be applied to degrade ECM and PNNs *in vitro* and *in vivo* and a viable aggrecan knockout mouse has been developed (Rowlands et al., 2018), there is currently no method of artificially inducing ECM growth or PNN formation. As discussed above, removal of or interfering with normal microglia function results in more highly condensed, intense, or concentrated PNNs (Liu et al., 2021; Barahona et al., 2022), but does not appear to result in *de novo* synthesis of the structures. To this end, the advent of a true PNN synthesis method would be a significant step towards truly confirming and/or revealing the roles of these structures in healthy and diseased brains alike.

One of the other stumbling blocks in determining the purposes and characteristics of PNNs and ECM in general is the fact that so many molecules comprise these complex structures that it becomes difficult to study. However, efforts to analyze and replicate its complexity have resulted in widely used biomaterials like basement membrane-like matrix (Matrigel) (Benton et al., 2011) and a variety of ECM-based polymers used for 3D modeling [see (Vigier, 2016) for a thorough review].

In addition to therapeutically targeting PNNs and the ECM to treat disorders such as epilepsy and AD, some suggest that manipulating these structures may be a potential anti-aging technique (Yang et al., 2021). As discussed previously, removal of PNNs using ChABC or hyase can restore the plasticity of the brain to critical period-like levels, implicating careful “editing” of the brain as a way to potentially mitigate or rewind the effects of age on memory formation and retention.

## 12 Summary

Epilepsy is a complex neurodegenerative disorder characterized by spontaneous, recurrent seizure activity, often expressed differently in every individual who suffers from it. The understanding of this disorder and its underlying causes is progressing, but it is inherently intricate and there are likely untold number of variables that contribute to epileptogenesis. Although neuroinflammation is only a part of the whole picture, we propose that astrocyte-PNN interactions both contribute to and result from neuroinflammation, thus exacerbating and enhancing epilepsy pathology and providing both a novel perspective as well as a potential therapeutic direction.

## Abbreviations

AD, Alzheimer disease; ADAMTSs, a disintegrin and metalloproteinase with thrombospondin motifs; AMPA, α-amino-3-hydroxy-5-methyl-4-isoxazolepropionic acid; CA1, CA2, cornu ammonis 1 and 2; CCL, chemokine C-C motif ligands; ChABC, chondroitinase ABC; CSPG, chondroitin sulfate proteoglycan; ECM, extracellular matrix; ECS, extracellular space; EEG, electroencephalogram; EPSC, EPSP, excitatory post-synaptic current or potential; FSN, fast-spiking neuron; GABA, γ-aminobutyric acid; HA, hyaluronic acid; Hapln, hyaluronan and proteoglycan link; HS, hippocampal sclerosis; IL, interleukin; LPS, lipopolysaccharide; MMP, matrix metalloproteinase; MTLE, medial TLE; PNN, perineuronal net; PTZ, pentylenetetrazol; PV, parvalbumin; TBI, traumatic brain injury; TLE, temporal lobe epilepsy; TLR, toll-like receptor; TNF-α, tumor necrosis factor α; WFA, Wisteria floribunda agglutinin.
